# Supplementary material for: First Molecular Evidence of Babesia caballi and Theileria equi in Imported Donkeys from Kyrgyzstan
Source: Pathogens. 2024 Aug 23;13(9):713. doi: 10.3390/pathogens13090713 (PMC11434632; doi:10.3390/pathogens13090713)
Supplement: Supplementary file 1 [file pathogens-13-00713-s001.zip › pathogens-3129411-supplementary.pdf]

Appendix Table S1. The PCR equipment was a Mastercycler X50s, Eppendorf, Germany.

1. PCR amplification to detect piroplasms based on the *18S rRNA*, *EMA-1* and *rap-1* gene

For all fragments, the reactions were performed in a final volume of 25 µL containing 12.5 µL of Premix *Taq* DNA polymerase (TakaRa, China), 1.0 µM of each primer and 1.0 µl of DNA template. If there was a second PCR, the reaction mixture was the same as the first PCR, except that the template was replaced by 1 µL of the first PCR product. The PCR cycling conditions were as shown in the following table.

***2.* Characteristics of amplified fragments and corresponding primer sequences**

Primer name	Gene	Primer sequence (5'-3)	Fragment	Cycling conditions of PCR assays	Reference
RLB(out)	*18S rRNA*	RLB-F2 (GACACAGGGAGGTAGTGACAAG)<br>RLB-R2 (CTAAGAATTTCACCTCTGACAGT)	400bp	95 °C for 3 min, followed by 35 cycles at 95 °C for 60 s , 52 °C for 50 s and 72 °C for 1.5 min, with a final extension at 72 °C for 5 min.	[1]
RLB(in)		RLB-F2 (GACACAGGGAGGTAGTGACAAG)<br>RLB-FINT (GACAAGAAATAACAATACRGGGC)		The second PCR 95 °C for 3 min, followed by 35 cycles at 95 °C for 30 s, 50 °C for 30 s and 72 °C for 30 s, with a final extension at 72 °C for 5 min.	[1]
EMA-1	*EMA-1*	EMA-1F (GCATCCATTGCCATTTCGAG)<br>EMA-1R (TGCGCCATAGACGGAGAAGC)	750bp	96°C for 10 min, followed by 40 cycles at 94°C for 60 s, 60.5°C for 60 s, and 72°C for 60 s, with a final extension at 72°C for 10 min.	[2]
rap-1	*rap-1*	rap-1F(CCAACCGCTGACCCTTC)<br>rap-1R (CTTCAGCTTCATGTACCACTTCTT)	568bp	95°C for 5 min, followed by 40 cycles at 95°C for 30 s, 56°C for 30 s, and 72°C for 60 s, with a final extension at 72°C for 7 min.	[3]

[1] Liu, J., Yang, J., Guan, G., Liu, A., Wang, B., Luo, J., Yin, H., 2016. Molecular detection and identification of piroplasms in sika deer (*Cervus nippon*) from Jilin Province, China. Parasit Vectors 9, 156. https://doi.org/10.1186/s13071-016-1435-3

[2] Alhassan, A., Pumidonming, W., Okamura, M., Hirata, H., Battsetseg, B., Fujisaki, K., Yokoyama, N., Igarashi, I., 2005. Development of a single-round and multiplex PCR method for the simultaneous detection of *Babesia caballi* and *Babesia equi* in horse blood. Vet Parasitol 129, 43–49. https://doi.org/10.1016/j.vetpar.2004.12.018

[3] Otgonsuren, D., Amgalanbaatar, T., Narantsatsral, S., Enkhtaivan, B., Munkhgerel, D., Zoljargal, M., Davkharbayar, B., Myagmarsuren, P., Battur, B., Battsetseg, B., Sivakumar, T., Yokoyama, N., 2024. Epidemiology and genetic diversity of *Theileria equi* and *Babesia caballi* in Mongolian horses. Infect Genet Evol 119, 105571. https://doi.org/10.1016/j.meegid.2024.105571
